# CAPTCHA Image Generation: Two-Step Style-Transfer Learning in Deep Neural Networks †

**DOI:** 10.3390/s20051495

**Published:** 2020-03-09

**Authors:** Hyun Kwon, Hyunsoo Yoon, Ki-Woong Park

**Affiliations:** 1 Department of Electrical Engineering, Korea Military Academy, Seoul 01805, Korea; hkwon.cs@gmail.com or; 2 School of Computing, Korea Advanced Institute of Science and Technology, Daejeon 34141, Korea; hyoon@kaist.ac.kr; 3 Department of Computer and Information Security, Sejong University, Seoul 05006, Korea

**Keywords:** machine learning, neural network, image style transfer, CAPTCHA, convolutional neural network (CNN)

## Abstract

Mobile devices such as sensors are used to connect to the Internet and provide services to users. Web services are vulnerable to automated attacks, which can restrict mobile devices from accessing websites. To prevent such automated attacks, CAPTCHAs are widely used as a security solution. However, when a high level of distortion has been applied to a CAPTCHA to make it resistant to automated attacks, the CAPTCHA becomes difficult for a human to recognize. In this work, we propose a method for generating a CAPTCHA image that will resist recognition by machines while maintaining its recognizability to humans. The method utilizes the style transfer method, and creates a new image, called a style-plugged-CAPTCHA image, by incorporating the styles of other images while keeping the content of the original CAPTCHA. In our experiment, we used the TensorFlow machine learning library and six CAPTCHA datasets in use on actual websites. The experimental results show that the proposed scheme reduces the rate of recognition by the DeCAPTCHA system to 3.5% and 3.2% using one style image and two style images, respectively, while maintaining recognizability by humans.

## 1. Introduction

With the advent of the Internet, millions of computers became connected, and various applications and services also became available through data collection and analysis. Moreover, with the development of various sensors, it became possible to collect information about the physical world. These sensors are usually networked with each other to collect, process, and transmit information. In particular, equipment such as mobile devices collect information using sensors or provide web services to users. Since mobile devices are connected to the Internet, they can be damaged by automated attacks such as DDoS attacks [1], spam messages [2], unlimited subscriptions [3], and bulletin boards [4] on websites. Therefore, it is becoming increasingly important to develop security solutions to defend against automated attacks on mobile devices. Completely automated public Turing test to tell computers and humans apart (CAPTCHA) [5] is one of the many security solutions for defense against such automated attacks. Through challenge-response tests, CAPTCHAs are used to determine if a user is a machine or a human. The method asks a question that is comprehensible by humans but not by machines. If the answer provided by humans is correct, the concerned service is provided, and if the answer is incorrect, the service is declined.

To create text-based CAPTCHA images, it is necessary to incorporate some distortion such as changing the size, curvature, and/or rotation of the text in images [6] so that they will be misrecognized by machines. Such distortion makes the images hard for a machine to recognize, but they are still understandable by humans. CAPTCHAs can also be based on audio or non-text images. Audio-based CAPTCHAs [7] provide numbers and letters in the form of sounds, with noise added so that machines will not understand them. The disadvantages of the audio method are that it can be interrupted by external sounds, and it requires an additional voice recognition system. In this study, we investigated CAPTCHA images that are based on text.

Methods of attack [8] for breaking CAPTCHAs have also been studied. To evade optical character recognition (ORC) attacks, it is necessary to modify the position, rotation, and/or size of the letters. However, if these distortions are too severe, it becomes difficult for humans to recognize the characters. Therefore, the goal is to make a CAPTCHA image that will be misrecognized by a machine but remains within the range of human recognition.

In the domain of machine learning, deep neural networks (DNNs) [9] have provided excellent service for image generation [10], image classification [11], and image synthesis [12]. In particular, convolutional neural networks (CNNs) [13] show good performance in the image recognition domain. With CNNs, it is possible to take out a representation of each feature and synthesize different images. The style transfer method [14], for example, can draw out a representation of the style of a different image and that of the content of an original image. This method alters the style of the image while retaining the original CAPTCHA, and has little effect on human perception.

In this study, we propose a method for generating a style-plugged-CAPTCHA image designed to resist recognition by machines while maintaining recognizability to humans, by applying style transfer learning in the CAPTCHA domain. The proposed method generates a CAPTCHA image by extracting the style feature of a different CAPTCHA and the content feature of the original CAPTCHA. This work is an extension of our previous study [15], in which we proposed the concept and principle of our method. A preliminary version of this article was presented at WISA 2019. The contributions of this study are as follows:

- Existing CAPTCHA generation methods assume that only humans can correctly recognize text images after distorting the characters or letters by overlapping, rotating, font, acr, etc. Compared to existing methods, the proposed style-plugged-CAPTCHA method has a unique characteristic of synthesizing distortions generated from several CAPTCHAs. We systematically describe the framework of the proposed method, and show that CAPTCHA images of various styles can be generated by applying multiple image styles to the original image.
- We analyze the degree of distortion in the images generated by the proposed scheme using CAPTCHA image datasets that operate on actual websites. In addition, we report the performance of the proposed method as measured by dividing the results for the case of one style image by that for the case of two style images. A human recognition test was conducted using the proposed method to analyze the human recognition rate.
- We report the rates of recognition by the DeCAPTCHA system for the proposed CAPTCHA images compared with those of the original images to verify the performance of the proposed method. (The CAPTCHA images used in the experiment are considered realistic as they are used in actual websites.)

The remainder of this paper is organized as follows: Section 2 reviews related literature. Section 3 presents the proposed method. Section 4 presents and explains the evaluation and the experiment. Section 5 discusses the proposed method, and Section 6 concludes the article.

## 2. Related Work

This section provides an overview of CAPTCHAs, the CNN model, and image transfer learning. The CAPTCHA method [16] was first introduced in AltaVista, which is an Internet search site. Section 2.1 provides an overview of CNNs. The image style transfer method is discussed in Section 2.2. Section 2.3 presents a review of text-based CAPTCHAs and Section 2.4 presents other methods for creating CAPTCHAs.

### 2.1. CNN Model

CNNs are used in dynamic tool identification [17] or human activity recognition [18]. The CNN [13] framework is a regularized version of a fully connected network, which is commonly used for visual imagery as a DNN. CNNs are advantageous in the sense that they reflect spatial information without any loss. CNNs can maintain spatial information because the output and input data are processed three-dimensionally. However, if only one-dimensional data is processed in the fully connected network, the spatial information for the three-dimensional data is lost.

**Figure 1:**
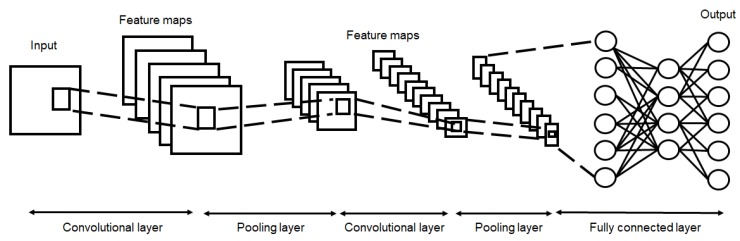
Architecture of the convolutional neural network (CNN) model.

Structurally, a CNN consists of a convolution layer, one or more fully connected layers, and one or more pooling layers, as shown in Figure 1. The characteristics of the image can be extracted through a filter in the convolution layer. The value of the output data can be obtained by multiplying the adjacent pixels through the convolution filter at each convolution layer. The value of the output data are the feature maps at each convolution layer. A fully connected layer is a layer which combines all the neurons of the previous layer. A pooling layer is used when subsampling is to be performed, or data samples are to be extracted through the convolution process. Like the convolution layer, a pooling layer uses only the adjacent pixel values, but no computation is performed. There are two types of pooling: average pooling and max pooling. Average pooling sets the average value of adjacent pixels, and max-pooling sets the largest pixel value of adjacent pixels.

The parameters for CNN are the filter window size, the number of convolution filters, the padding, and the stride. In the intermediate stage where several small filters overlap, the filter window size can be utilized by using the non-linearity to emphasize the features. Regarding the convolution filters, it is important to maintain a relatively constant number of filters for each layer. In order to prevent information loss and to adjust the number of output data, the padding value increases the number of input data around a specific pixel value. The stride can control the distance moved by the window.

### 2.2. Image Style Transfer Method

Image style transfer technique [14] creates a new image by combining the content of the original image with the style of another image. This technique uses a feature of CNNs that can take out information from the semantic image at a high level. It can keep the specific content by increasing the content information of the original image. For example, let’s say that a picture of Obama is the original image, and a Van Gogh style painting is the style image. Using the image style transfer method, an Obama picture drawn in the Van Gogh style can be created. Various image syntheses can thus be produced. The style transfer method minimizes two loss functions: the style information about the feature space of the other image and the content information of the original image. The proposed scheme is a technique for applying this image style transfer method to CAPTCHA images.

### 2.3. Text-Based CAPTCHA

Text-based CAPTCHAs are distorted text images that will be misrecognized by machines but correctly recognized by humans. To defend against a machine recognizing a CAPTCHA, text-based CAPTCHAs may overlap the letters or the letters, rotate the text image, or add curvature to the text image. Typically, text-based CAPTCHAs are generated using one of three methods. The first is the hollow-CAPTCHA method [19] which connects all text together. This method can have resistance to recognition and segmentation by machines, while enhancing perceptibility by humans. The second is the connecting characters together (CCT) method [20], which adds overlapping and noise to characters, and adds curvature to text images in order to increase resistance to recognition and character segmentation via automated attacks. The third is the character isolation method [21], which displays each character independently of the others, unlike the other two methods. With this method, the distortion of each character is more severe than with the other techniques.

However, if too much distortion is applied, text-based CAPTCHAs are disadvantageous in the sense that they degrade recognizability by humans. Unlike these conventional methods, the proposed scheme applies different styles to images while maintaining human recognizability.

### 2.4. Other Methods for Generating CAPTCHAs

There are various methods for generating CAPTCHA images. One of them is the generative adversarial net method (GAN) [22]. GAN combines several CAPTCHA images to create a new CAPTCHA image. This method uses the distortion variable to generate a CAPTCHA that the machine misrecognizes through the zero-sum game method. GAN was also tested using actual CAPTCHA images. The second method [23] generates CAPTCHA images using the adversarial example technique. Adversarial example adds a little noise to the original CAPTCHA to maintain human perception, while CAPTCHA recognition systems misidentifies it. This method is characterized to avoid attack by manipulating the test data against the pre-trained CAPTCHA system. The third method is the combination of grid-CAPTCHA and font-CAPTCHA [24]. The grid-CAPTCHA method selects the relevant CAPTCHA image related to the provided sentence. The font-CAPTCHA method suggests adding Chinese characters to the image, and then clicking the characters in the order of Chinese characters by using image transfer learning. Similarly, various other methods for generating CAPTCHA images have been introduced.

## 3. Proposed Scheme

This section gives an overview of the proposed method. The proposed scheme can create a new CAPTCHA by compositing one or two style images with a content image. In this method, the merge process is performed in steps according to the number of style images. The method has the advantage of creating a new CAPTCHA by incorporating distortions reflected in one or two style images.

### 3.1. Process for Transferring the Style Image to the Original Image

**Figure 2:**
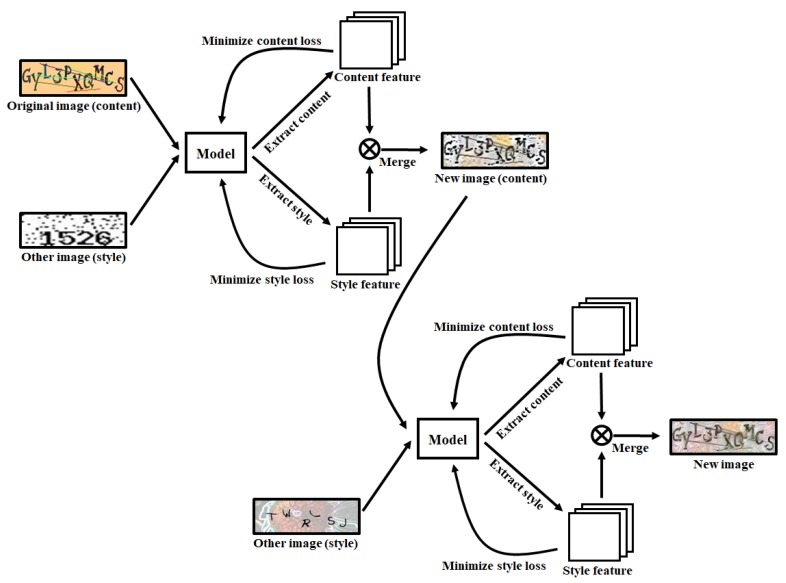
Architecture of the proposed method.

To create a CAPTCHA image, the proposed scheme takes the original image and a second image as input values, and creates a new CAPTCHA image that incorporates the content of the original CAPTCHA with the style of the second CAPTCHA, as shown in Figure 2.

The proposed method uses the style transfer method [14]. In this method, the style feature is extracted from another image, and the content feature is extracted from the original image separately. For the content feature, the convolution layer of the original image can extract a feature map. As the depth of the layers increases, the semantic information about the content image remains, but pixel-level information disappears. Thus, from one of the deep layers, the method can extract the content feature of the original image. The style feature uses the Gram matrix [25], which represents the correlations between the feature maps of each layer. Through the correlations of the feature maps of each layer, multiple scales of stationary information can be obtained, rather than the layout information for the image. The greater the number of deep layers that are included, the more static information about the image is obtained, and the less layout information generated.

To create the composite CAPTCHA image, the proposed scheme modifies the noise image $\vec{x}$ by using back-propagation of the loss function $loss_{T}$. The loss function $loss_{T}$ is the sum of style loss $loss_{\mathrm{style}}$ and content loss $loss_{\mathrm{content}}$:

(1) $$loss_{T}=loss_{\mathrm{style}}\left(\vec{a},\vec{x}\right)+\alpha \cdot loss_{\mathrm{content}}\left(\vec{p},\vec{x}\right).$$

Here, $\vec{a}$ is the second image; $\vec{x}$ is a noise image, which is a composite image; $\vec{p}$ is the original image; and α is the content loss weighted value greater than or equal to 1 whose initial value is 1.

The style loss $loss_{\mathrm{style}}$ is obtained based on the noise image $\vec{x}$, and the style feature for the second image (style) $\vec{a}$. The detailed steps are as follows: first, the noise image $\vec{x}$ and the second image $\vec{a}$ are each fed forward through the neural network. Second, we can calculate Gram matrices *A* and *G* in the layer *l* as input values for the noise image $\vec{x}$ and the second image $\vec{a}$, respectively. Third, the style loss is calculated using these Gram matrices *A* and *G*, as follows:

(2) $$E_{l}=\frac{1}{4N_{l}^{2}M_{l}^{2}}\sum_{i,j}{\left(G_{ij}^{l}-A_{ij}^{l}\right)}^{2},$$

where $A_{ij}^{l}$ and $G_{ij}^{l}$ are the inner products between the vectorized feature maps *i* and *j* in layer *l*, $N_{l}$ is the number of feature maps at layer *l*, and $M_{l}$ is the width × height of the feature maps at layer *l*. For the style feature, the total style loss $loss_{\mathrm{style}}$ is as follows:

(3) $$loss_{\mathrm{style}}\left(\vec{x},\vec{a}\right)=\sum_{l=0}^{L}w_{l}E_{l},$$

where $w_{l}$ is the weighting factor of layer *l* in the total loss.

The content loss $loss_{\mathrm{content}}$ is obtained based on the content information for the noise image $\vec{x}$, which is incorporated with the original image $\vec{p}$. The detailed steps are as follows: First, the noise image $\vec{x}$ and the original image $\vec{p}$ are each fed forward through the neural network. Second, we can calculate feature maps *F* and *P* in layer *l*, as input values for the noise image $\vec{x}$ and the original image $\vec{p}$, respectively. Third, the content loss is calculated using the feature maps *F* and *P* as follows:

(4) $$loss_{\mathrm{content}}\left(\vec{x},\vec{p},l\right)=\frac{1}{2}\sum_{i,j}{\left(F_{ij}^{l}-P_{ij}^{l}\right)}^{2},$$

where $P_{ij}^{l}$ is the *i*th activation filter at position *j* in layer *l* and $F_{ij}^{l}$ is the *i*th activation filter at position *j* in layer *l*. The details of the procedure for transferring the style image to the original image are given in Algorithm 1.
**Algorithm 1** Transferring the style image to the original image. **Input:**$P_{ij}^{l}$, the *i*th activation filter at position *j* in layer *l*; $F_{ij}^{l}$, the *i*th activation filter at position *j* in layer *l*; $A_{ij}^{l}$ and $G_{ij}^{l}$, the inner products between the vectorized feature maps *i* and *j* in layer *l*; $N_{l}$, the number of feature maps at layer *l*; $M_{l}$, the height × width of the feature maps at layer *l*; iterations *r*, noise image $\vec{x}$; original image $\vec{p}$; second image (style) $\vec{a}$.** The process of transferring the style image to the original image:** $\vec{a}\leftarrow 0$ **for** r step **do**  $loss_{\mathrm{content}}\left(\vec{x},\vec{p},l\right)\leftarrow \frac{1}{2}\sum_{i,j}{\left(F_{ij}^{l}-P_{ij}^{l}\right)}^{2}$  $E_{l}\leftarrow \frac{1}{4N_{l}^{2}M_{l}^{2}}\sum_{i,j}{\left(G_{ij}^{l}-A_{ij}^{l}\right)}^{2}$  $loss_{\mathrm{style}}\left(\vec{x},\vec{a}\right)\leftarrow \sum_{l=0}^{L}w_{l}E_{l}$  $loss_{T}\leftarrow \alpha \cdot loss_{\mathrm{content}}+loss_{\mathrm{style}}$  Update $\vec{x}$ by $\mathrm{minimizing}\;loss_{T}\;$ through back-propagation such as by $\;\vec{a}-\lambda \frac{\delta loss_{T}}{\delta \vec{\vec{a}}}$ **end for** $return\;\vec{a}$

### 3.2. CAPTCHA Creation Process with Two Style Images

The proposed method creates a CAPTCHA with a new style by following the steps above. As shown in Figure 2, when there is one original image and two style images, a new image is first created by using one of the style images. Then, the newly created image becomes the original image and is combined with the other style image to create the final image.

## 4. Experiment and Evaluation

This section evaluates the performance of the proposed scheme and shows the experimental results. In our experiments, the proposed scheme was used to create CAPTCHA images that have resistance to DeCAPTCHA, and maintain their recognizability to humans. We used the TensorFlow [26] library, which is an open-source machine learning library.

### 4.1. Experimental Methods

This subsection describes the pre-trained model and the parameters used in the proposed method: the CAPTCHA image dataset, and the DeCAPTCHA system. The proposed scheme used six CAPTCHA datasets, each running on a different website, and 100 data elements per dataset. The six websites are: nationalinterest.org, mail.aol.com, cesdb.com, smart-mail.de, tiki.org, and articlesfactory.com.

For the DeCAPTCHA and the pre-trained model, we used the GSA captcha breaker program [27], which is the software used on the websites for recognizing and segmenting CAPTCHA images as DeCAPTCHA. We used the VGG-19 model [28] as a pre-trained model. Table 1 and Table 2 show the structure and the parameters for the VGG-19 model.

L-BFGS optimization [29] was used as box-constrained optimization to create the style-plugged CAPTCHA. The L-BFGS algorithm can solve large-scale problems by using quadratic optimization functions. The number of iterations was 100, and the content loss weight α, was 1. The proposed scheme updated the output $\vec{x}$ by minimizing the style and content loss for the given number of iterations. The new image, $\vec{x}$, was analyzed in terms of perceptibility by humans, and the rate of recognition by DeCAPTCHA at the end of the iterations. In cases with two style images, the new image was regarded as a content image again, and the above process was repeated by compositing it with the new style image. The rate of recognition by DeCAPTCHA is the rate at which the CAPTCHA is correctly recognized by the GSA breaker system.

### 4.2. Experimental Results

The experimental results were analyzed by dividing the difference in the style-plugged CAPTCHA generated using one style image by using two style images. We show samples of CAPTCHAs generated from one style image and from two style images. In addition, we analyze the rate of recognition by the DeCAPTCHA system on the CAPTCHA images generated by the proposed method.

#### 4.2.1. One Original Image and One Style Image

**Figure 3:**
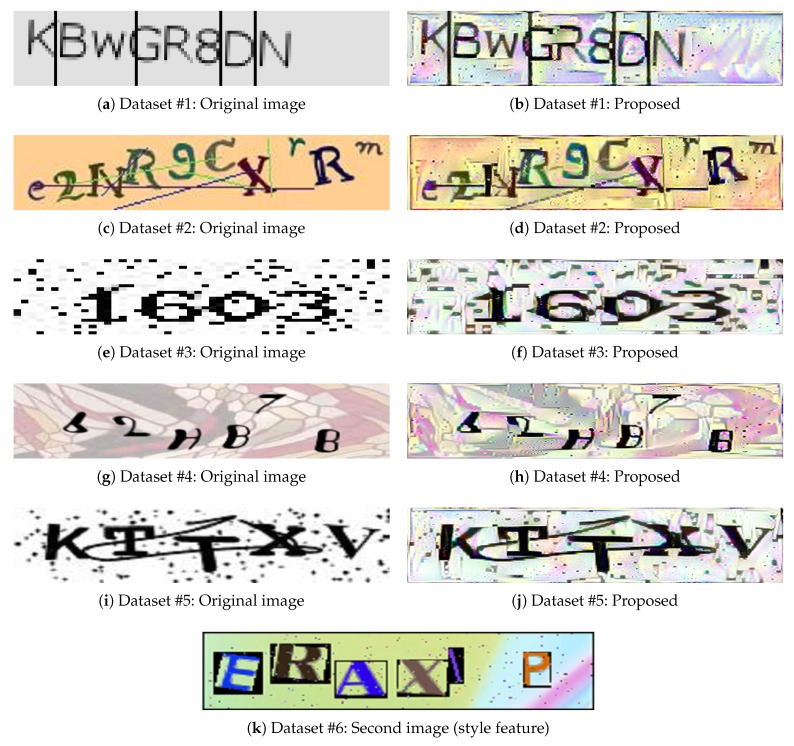
Examples of style-plugged CAPTCHAs formed using one style image. One original image from each dataset is shown, and an image from dataset #6 was used as the style image. “Proposed" is style-plugged CAPTCHA.

This subsection shows the experimental results obtained when one style image was applied to one original image. Figure 3 shows examples of the proposed CAPTCHA images (style-plugged-CAPTCHA images) generated from an original image taken from each dataset, when an image from the dataset #6 was applied as the style image. The proposed CAPTCHA images in the figure were generated by taking the style properties of the second image, while maintaining the content properties of the original image. Although the style image used was the same, the degree of deformation in the style differed slightly based on the characteristics of the original image. However, it can be seen that the rate of recognition by humans is retained because many of the letters are not transformed in terms of their perceptibility by humans.

**Figure 4:**
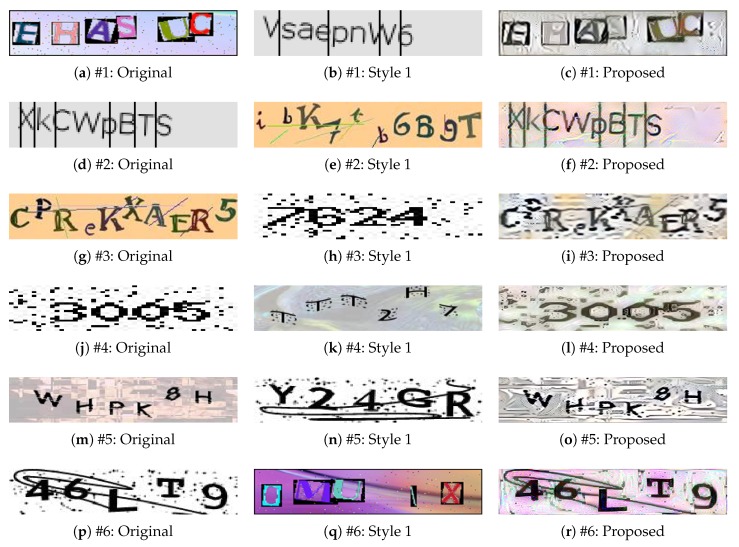
Examples of style-plugged CAPTCHAs formed using one style image. “Original" is the original image, “Style 1" is the second image, and “Proposed" is the style-plugged-CAPTCHA image.

Figure 4 shows CAPTCHA images created for each dataset when the original sample and the second image are incorporated. The CAPTCHA images in the figure were created by extracting the style of the second image while retaining the content of the original sample. In particular, the datasets #3–#6 show that the dotted quality of the style image has been inserted in the CAPTCHA images. In addition, it can be seen that the newly generated CAPTCHA images remain recognizable to humans.

#### 4.2.2. One Original Image and Two Style Images

**Figure 5:**
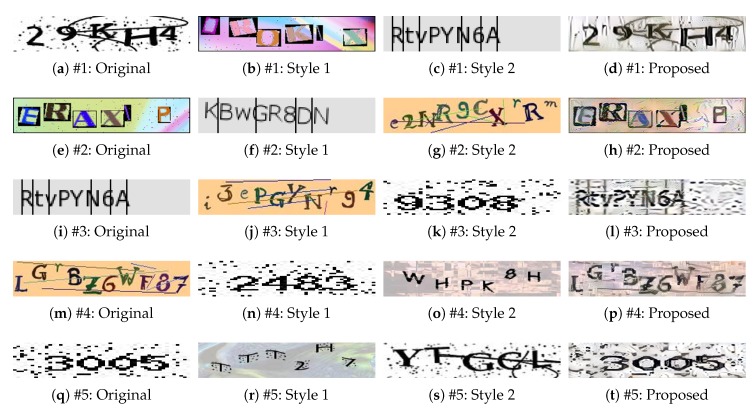
Examples of style-plugged CAPTCHAs formed using two style images. A set of images for each dataset is shown: “Original" is the original image, “Style 1" is style image 1, “Style 2" is style image 2, and “Proposed" is the style-plugged-CAPTCHA image.

This subsection shows the experimental results obtained when two style images were applied to one original image. Figure 5 shows the CAPTCHA images created by the proposed method when the content of the original image and two different style images were synthesized. The figure shows that the contents of the original samples were preserved, and the styles of style image 1 and style image 2 were imported. In case #4, for example, the style-plugged-CAPTCHA image shows that the dotted quality was taken from style image 1 and the image color was imported from style image 2. In general, the style-plugged-CAPTCHA images reflect many style elements corresponding to style image 2. This is because the style of the style image that is applied last in the creation process will have the most influence. In this way, style-plugged-CAPTCHA images with various styles can be generated from the original image.

**Figure 6:**
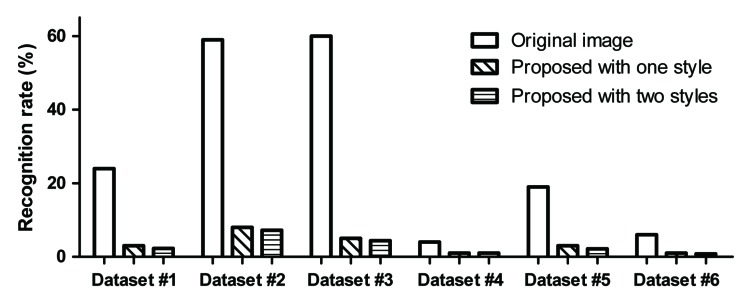
Rates of recognition by DeCAPTCHA. “Proposed" is style-plugged CAPTCHA.

Figure 6 shows the rates of recognition by the DeCAPTCHA program for the original images and the proposed CAPTCHAs generated using one style image and using two style images, based on 100 samples per dataset. As can be seen in the figure, the rate of recognition by DeCAPTCHA is different for each dataset. Note that when the proposed method is applied to generate CAPTCHA images, the rate of recognition by DeCAPTCHA is significantly lower for these images owing to the modulation of the style. It can also be seen that the proposed CAPTCHA images generated using two style images have a lower recognition rate. This is because incorporating two style images lead to more distortion. Thus, the CAPTCHA images produced using the proposed method have somewhat more resistance to the DeCAPTCHA system than the original images.

#### 4.2.3. Human Recognition

**Figure 7:**
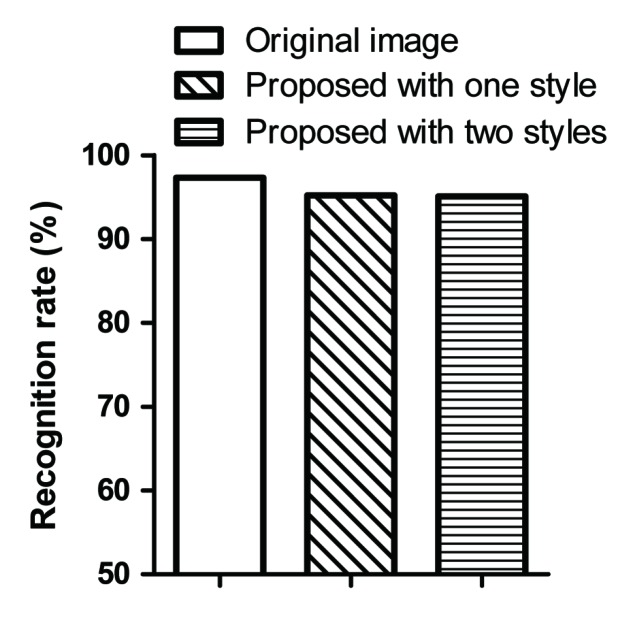
Rates of recognition by humans. “Proposed" is style-plugged CAPTCHA.

This subsection shows the results of the human recognition test on the CAPTCHA images generated by the proposed method and the original CAPTCHA images. It is important to maintain human recognition in CAPTCHA images. Therefore, it is necessary to analyze the human recognition rate for the proposed method. Figure 7 shows the rates of human recognition for the original images and the CAPTCHA images generated using one style and two style images. Human testing was conducted with 10 researchers from Sejong University, and 100 random sets of images were tested. As can be seen in Figure 7, the rates of human recognition of the original images and the generated CAPTCHA images are nearly the same. The reason for the low recognition rate was human misrecognition due to the presence of too much distortion in the original images. Therefore, the proposed method maintains a level of recognizability to humans that is nearly the same as that of the original image.

## 5. Discussion

This section discusses the style images, the attack method considerations, human recognition, applications, and limitations for the proposed scheme.

### 5.1. Style Images

The proposed scheme can create a variety of CAPTCHA images according to the number of style images used, by applying the styles to the CAPTCHA images using a cascaded ensemble method. However, at each stage, the style of the last image will be the one that is the most apparent in the generated CAPTCHA image. Therefore, multiple styles can be applied when creating CAPTCHAs, and the most heavily weighted style image needs to be applied at the end.

In addition, the proposed method shows how to create a CAPTCHA that maintains the content of the original image using one or two style images. This method can be extended to create combined CAPTCHA images of three or more style images.

### 5.2. Attack Method Considerations

The assumption underlying the proposed method is that an attack will be a white box attack by an attacker that knows the pre-trained model. The proposed scheme is a way of drawing out the content feature of the original CAPTCHA and the style feature of a second CAPTCHA.

The proposed scheme gives more weight to the content of the original CAPTCHA. Because the rate of recognition by humans decreases when there are many modifications to the content of the original CAPTCHA, the weight for the content representation is set higher than that for the style representation. If we give less weight to the original content loss ($loss_{\mathrm{content}}$), the content of the original CAPTCHA may be distorted or corrupted. Therefore, the content loss weight α is greater than or equal to 1, as given by Equation (1).

(5) $$loss_{T}=loss_{\mathrm{style}}\left(\vec{a},\vec{x}\right)+\alpha \cdot loss_{\mathrm{content}}\left(\vec{p},\vec{x}\right).$$

The reason for choosing the initial value as 1 is to show that the content part is sufficiently preserved, even if the content loss weight α is 1. To better preserve the content of the original CAPTCHA, the content loss weight α can be chosen to be greater than 1.

Unlike conventional CAPTCHA generation methods, the proposed technique changes the style by using the capability of CNNs to extract features. A wider variety of CAPTCHA images can be generated by changing the image style, rather than by modifying the characters. In particular, multiple style images can be combined to create new CAPTCHA images.

### 5.3. Human Recognition

Maintaining human recognition is important, as human users are not provided with the concerned service if the human recognition rate drops. However, if a CAPTCHA is too easily identifiable by humans, then machines can become aware of an automated attack. In addition, to evade such attacks, it is necessary to modify the position, rotation, and/or size of the letters, but if too severe an adjustment is made, it will be difficult for the characters to be recognized by humans. Therefore, the goal is to make a CAPTCHA image that will be misrecognized by a machine but remains within the range of human recognition. In this regard, the proposed method applies multiple image styles to create a CAPTCHA that is difficult for machines to recognize, while maintaining the recognition rate of the original CAPTCHA.

In addition, as the content loss weight increases, human perception increases due to greater preservation of the content. Total loss consists of content loss and style loss, as given by Equation (1). If we put more weight on content loss, the new CAPTCHA image will be less reflective of the image style. Moreover, if we put less weight on content loss, the content of the new CAPTCHA may be distorted or corrupted.

**Figure 8:**
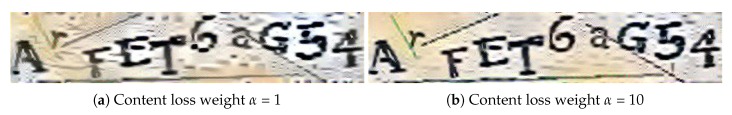
Examples of style-plugged CAPTCHAs according to the content loss weight α.

Figure 8 shows examples of style-plugged CAPTCHAs according to the content loss weight α. It can be seen in the figure that as the content loss weight increases, the content is preserved more, while the effect on the style image decreases.

### 5.4. Applications

The proposed technique is useful for creating CAPTCHA images in large quantities. If there are not enough CAPTCHA images, it is necessary to create more CAPTCHA images by combining a variety of other images. In this situation, the proposed technique can be used to create CAPTCHA images of different styles. In addition, if a DeCAPTCHA system needs to be trained on a variety of data, the proposed method can be used to enhance the performance of the DeCAPTCHA system by creating a variety of images and allowing learning in advance.

### 5.5. Limitations

It is necessary to consider the selection of a style image. In our method, if the weight of the style of the second image is increased, the image distortion may be increased. If the second image (style image) is severely distorted, the final image may be affected. In addition, it is necessary to compare the original CAPTCHA with the generated CAPTCHA in order to check human recognizability.

## 6. Conclusions

The proposed scheme can create style-plugged-CAPTCHA images that change the style of the original CAPTCHA images. The proposed technique generates a CAPTCHA image by drawing out the style feature of a second image, while retraining the content feature of an original sample. The CAPTCHA images generated by our method have a degree of resistance to DeCAPTCHA systems. The experimental results show that the proposed scheme retains the rate of human recognition while decreasing the rate of recognition by the DeCAPTCHA system to approximately 3.5% and 3.2% using one style image and two style images, respectively. The proposed scheme also has the potential for use in applications such as data expansion.

Future research will be an interesting topic in terms of data and generation. In future studies, the proposed method can be extended to the voice or natural image selection domains. Generating CAPTHAs using GAN is also an intriguing future prospect.

## Figures and Tables

**Table 1:** Model of VGG-19 [28]. “Con" is a convolution layer. “R" is the ReLU activation. “Max" is a Max pooling. “Fully" is a fully connected layer.

Layer Type	Shape
Con + R	[3, 3, 64]
Con + R	[3, 3, 64]
Max	[2, 2]
Con + R	[3, 3, 128]
Con + R	[3, 3, 128]
Max	[2, 2]
Con + R	[3, 3, 256]
Con + R	[3, 3, 256]
Con + R	[3, 3, 256]
Con + R	[3, 3, 256]
Max	[2, 2]
Con + R	[3, 3, 512]
Con + R	[3, 3, 512]
Con + R	[3, 3, 512]
Con + R	[3, 3, 512]
Max	[2, 2]
Con + R	[3, 3, 512]
Con + R	[3, 3, 512]
Con + R	[3, 3, 512]
Con + R	[3, 3, 512]
Max	[2, 2]
Fully + R	[4096]
Fully + R	[4096]
Fully + R	[1000]
Softmax	[1000]

**Table 2:** Parameters of VGG-19 [28].

Contents	Value
Momentum	0.9
Learning rate	0.01
Decay rate	0.0005
Number of iterations	370,000
Number of epochs	74
Batch size	256
Dropout	0.5
